# Real-World Data for Lenvatinib in Radioiodine-Refractory Differentiated Thyroid Cancer (RELEVANT): A Retrospective Multicentric Analysis of Clinical Practice in Austria

**DOI:** 10.1155/2020/8834148

**Published:** 2020-11-28

**Authors:** G. Rendl, B. Sipos, A. Becherer, S. Sorko, C. Trummer, M. Raderer, W. Hitzl, M. Ardelt, H. J. Gallowitsch, C. Pirich

**Affiliations:** ^1^Department of Nuclear Medicine and Endocrinology, University Hospital Salzburg, Paracelsus Medical University Salzburg, Salzburg, Austria; ^2^Department of Nuclear Medicine, Academic Teaching Hospital Feldkirch, Feldkirch, Austria; ^3^Department of Nuclear Medicine and Endocrinology, PET/CT Centre, Klinikum Klagenfurt am Wörthersee, Klagenfurt, Austria; ^4^Division of Endocrinology and Diabetology, Department of Internal Medicine, Medical University of Graz, Graz, Austria; ^5^Department of Internal Medicine I, Division of Oncology, Medical University of Vienna, Vienna, Austria; ^6^Research Office (Biostatistics), Paracelsus Medical University Salzburg, Salzburg, Austria; ^7^Department of Ophthalmology and Optometry, University Hospital Salzburg, Paracelsus Medical University Salzburg, Salzburg, Austria; ^8^Research Program Experimental Ophthalmology and Glaucoma Research, University Hospital Salzburg, Paracelsus Medical University Salzburg, Salzburg, Austria; ^9^Institute of Pharmacy, Paracelsus Medical University Salzburg, Salzburg, Austria

## Abstract

**Background:**

Lenvatinib has proven efficacy in progressive, radioiodine- (RAI-) refractory thyroid cancer (TC). Dose reductions are commonly performed due to decreased tolerability and adverse effects. This retrospective multicenter study analyzed overall survival (OS) and progression-free survival (PFS) and tolerability in the Austrian patient population treated with lenvatinib.

**Methods:**

Clinical data of 43 patients (25 males and 18 females) with a median age of 70 years (range: 39–91 years) and RAI-refractory TC with metastases to the lymph nodes (74%), lungs (86%), bone (35%), liver (16%), and brain (12%) were analyzed. The mean duration of treatment with lenvatinib was 26.6 ± 15.4 months with dosage reductions required in 39 patients (91%).

**Results:**

PFS after 24 months was 71% (95% CI: 56–87), and overall survival (OS) was 74% (95% CI: 60–88), respectively. OS was significantly shorter (*p*=0.048) in patients with a daily maintenance dosage ≤ 10 mg (63%) (95% CI: 39–86) as compared to patients on ≥ 14 mg lenvatinib (82%) (95% CI: 66–98) daily. Dose reduction was noted in 39 patients (91%). Grade ≥3 toxicities (hypertension, diarrhea, weight loss, and palmar-plantar erythrodysesthesia syndrome) were most common leading to discontinuation of lenvatinib in 7 patients (16%).

**Conclusion:**

Lenvatinib showed sustained clinical efficacy in patients with metastatic RAI-refractory TC even with reduced maintenance dosages over years. The effects were comparable to the registration trial, although patients had a higher median age and, more commonly, dose reductions.

## 1. Introduction

Thyroid cancer (TC) is the most common endocrine malignancy, with a rising incidence worldwide. According to Statistik Austria, 822 cases of TC (9.2 per 100,000 people) were diagnosed in 2016 of whom 78 patients died (0.9 per 100,000 people) [1, 2]. Histopathologically, three types of TC exist: differentiated thyroid cancer (DTC), medullary thyroid cancer, and anaplastic thyroid cancer [3]. DTC represents the vast majority of thyroid cancer cases (>90%), including both papillary and follicular forms [3, 4]. In 2013, the percentage of the papillary form amounted to 84% among newly diagnosed TC within the federal state of Salzburg, Austria [5].

In terms of standardized treatment, surgical removal of the thyroid gland, i.e., thyroidectomy, followed by radioiodine ablation (RAI) and thyroid-stimulating hormone suppression therapy with levothyroxine has been the standard of care for all DTC, except for pT1a in Austria [6]. According to the guideline of the European Association of Nuclear Medicine, an ablation of any residual thyroid remnant tissue following thyroidectomy with RAI enables the follow-up of the patient by the use of the biomarker thyroglobulin and decreases the incidence of recurrent disease [7].

The majority of patients with DTC responds well to this multimodal standard treatment regimen; however, up to 15% of DTC cases develop RAI-refractory disease [8–10]. RAI-refractory disease is associated with an increased risk of death from DTC [8, 9], though its definition is not uniform among national and international guidelines and recommendations published [6, 9, 11, 12].

In a landmark retrospective study, patients with RAI-refractory disease exhibit a 10-year survival rate of only 10% from the time of detection of metastases [8, 9], therefore often requiring a multimodular treatment regimen which includes systemic therapies [11].

The therapeutic strategy for the treatment of radioiodine-refractory thyroid cancer has been changing with market approval and introduction of tyrosine kinase inhibitors (TKIs). The DECISION trial employing Sorafenib was first used to demonstrate a significant prolongation in progression-free survival (PFS) in radioiodine-refractory DTC [13].

Lenvatinib is an oral multitargeted TKI of vascular endothelial growth factor receptor (VEGFR) 1–3, fibroblast growth factor receptor (FGFR) 1–4, platelet-derived growth factor receptor- (PDGFR-) *α*, and RET and KIT proto-oncogenes [14, 15]. Lenvatinib was approved for the treatment of radioiodine-refractory DTC in the United States and Europe [16, 17] based on the results of the pivotal phase-3 SELECT study [18].

However, the benefit of systemic treatment with TKI must outweigh the potential harm of this class of drugs since a substantial proportion of patients might present without symptoms and with minor degree of disease progression for years [19].

Therefore, national guidelines have been introduced to define the patient population which might benefit most from the initiation of systemic treatment with the TKI [6, 12].

The aim of our retrospective multicenter study was to analyze (1) the Austrian patient population treated with lenvatinib after its European approval, (2) the efficacy, and (3) the tolerability and toxicity of this treatment with regard to the profile and severity of adverse effects and their impact on maintenance dose.

## 2. Materials and Patients

This study included 43 patients (18 female and 25 male patients) with a mean age of 67 years ±11 years (median age: 70 years; range: 39–91 years) from five nuclear medicine, endocrine, or oncologic departments in Austria located in Salzburg (*n* = 13), Feldkirch (*n* = 7), Klagenfurt (*n* = 11), Graz (*n* = 3), and Vienna (*n* = 9).

This study was approved by the leading ethics committee of the Federal Republic of Salzburg and, subsequently, if required, by the local ethics committee.

The age at initial diagnosis of thyroid carcinoma was 60 ± 13 years (median age: 63 years; range: 33–90 years).

The majority of patients had DTC (84%) (Table 1). Initial treatment consisted of thyroidectomy in 42 out of 43 patients, and 41 patients (95%) had at least 1 RAI therapy.

Eleven patients underwent postoperative local radiotherapy of the thyroid bed (26%), with a mean dose of 57 Gray (Gy) ± 7 Gy (median dose: 60 Gy; range: 45–66 Gy), and 12 patients (28%) had radiotherapy of metastases. Nine out of 12 patients had external beam radiation therapy of metastatic bone lesions, 4 patients of metastatic pulmonary lesions, 2 patients had palliative radiotherapy of lymph node metastases, and one patient of hepatic metastasis, respectively. No patient underwent local radiotherapy during lenvatinib treatment.

In all patients, treatment initiation with a TKI such as lenvatinib was based on progressive disease in imaging (RECIST 1.1 [20]; PERCIST [21]) and by subsequent approval by an interdisciplinary tumor board consisting of specialists in radiation oncology, nuclear medicine, surgery, pathology, and oncology [6].

Eight patients (19%) had prior TKI treatment, of whom six were on Sorafenib and two on pazopanib, respectively (Table 1). Progression of disease, mostly lung metastasis (86%), in imaging was the only cause for switching from conventional therapy or 1^st^-line TKI therapy to lenvatinib.

Mean duration of disease until initiation of lenvatinib with a starting dose of 24 mg daily was 7.1 ± 6.0 years (median: 5.9 years; range: 0.1–23.2 years), and mean patient age at start of lenvatinib was 67 ± 11 years (median: 70 years; range: 39–91 years).

### 2.1. Standard Clinical Care

Symptoms, blood pressure and vital signs, and laboratory testing including levels of thyroglobulin (TG), thyroid-stimulating hormone (TSH), and thyroglobulin antibodies (TgAb), as well as urinary analysis, were routinely recorded during follow-up, respectively.

After initiation of lenvatinib, follow-up visits were scheduled at 1-month intervals. Adverse effects were assessed according to the National Cancer Institute Common Terminology Criteria for Adverse Events (version 5) [22]. Dose reductions were performed due to treatment-related adverse effects at regularly scheduled follow-up visits. 3-month intervals were applied for patient monitoring in case of stable course of disease and their well-being.

Imaging was performed at 3- to 6-month intervals or in case of suspected progression of disease. Imaging consisted of computed tomography (CT) imaging in Vienna and Graz and F-18 fluorodeoxyglucose positron emission tomography/computed tomography (FDG PET/CT) imaging in all other centers.

In all centers, TG levels were evaluated 1, 3, 12, and 24 months after beginning of lenvatinib therapy, respectively.

Response to therapy was defined as the absence of tumor progression in imaging (RECIST 1.1 or PERCIST) and absence of clinical complications due to tumor progression.

### 2.2. Laboratory Methods

Standard laboratory methods were employed in all centers. All patients underwent standardized clinical workup with assessment of clinical status, body weight, blood pressure, and measurement of free thyroid hormones (free T3 and free T4), TG, and TgAb, electrolytes, albumin, liver and renal function, red and white blood cell, and platelet count. Standard urinary analysis was performed regularly, mainly for the assessment and course of micro- or macroproteinuria.

### 2.3. Data Collection and Statistical Analysis

Source data collection was performed at each center by the use of individual (electronic) patient records employing a standardized Microsoft Office Excel data sheet (Microsoft Corporation, Redmond, WH, USA). Data were anonymized and transferred for analysis to the statistical department of the Research Office of the Paracelsus Medical University Salzburg, Austria. Data were checked for consistency and normality. Fisher's exact test and Pearson's test were used to analyze cross-tabulations and distributions (age, sex, histology, and radioiodine therapies). One-factorial ANOVA and corresponding LSD test were used for pairwise comparisons. Kaplan–Meier analysis and Cox regression models were used for survival analysis. 95% confidence intervals were computed for survival analyses and thyroglobulin levels under therapy. All reported tests were two-sided, and *p* values <0.05 were considered statistically significant. All statistical analyses in this report were performed by the use of NCSS (NCSS 10, NCSS, LLC, Kaysville, UT) and Statistica 13 (Hill, T. and Lewicki, P. Statistics: Methods and Applications. StatSoft, Tulsa, OK).

## 3. Results

### 3.1. Survival

At the time of data cutoff (September 30, 2019), the mean duration of lenvatinib therapy was 26.6 ± 15.4 months (median: 27.6 months; range: 1.2–60.0 months) at a mean observation period of 32.6 ± 13.7 months (median: 34.6 months; range: 4.1–60.0 months) in all patients. Ten patients (one of them with prior TKI therapy) had died after a mean duration of 9.5 ± 7.5 months (median: 5.9 months; range: 1.5–21.3 months) of lenvatinib, whereas seven patients discontinued lenvatinib treatment after a mean duration of 21.1 ± 14.4 months (median: 21.6 months; range: 1.2–37.2 months). The remaining 26 patients were still on lenvatinib treatment at the time of data cutoff with a mean duration of 32.6 ± 13.7 months (median: 34.6 months; range: 4.1–60.0 months).

Overall survival (OS) after 24 months was 74% (95% CI: 60–88). The progression-free survival (PFS) after 24 months was 71% (95% CI: 56–87). Median OS and PFS has not been reached, yet.

The OS was not influenced by tumor subtype (FTC, PTC, or poorly differentiated/anaplastic TC) in our patient collective (*p*=0.66).

Five patients had brain metastases, of whom three died, with a median OS of 12 months (range: 1–22 months), and seven patients had liver metastases, of whom three also died, with a median OS of 32 months (range: 5–48 months). The median OS of the remaining 31 patients (4 of whom died) amounted to 26 months (range: 4–49 months).

PERCIST showed partial remission (PR) in 9% (*n* = 4), stable disease (SD) in 16% (*n* = 7), and progressive disease (PD) in 28% (*n* = 12) of the 43 patients. One patient died after 3 months without restaging FDG PET/CT after initiation of lenvatinib therapy. No data were given for the remaining 19 patients (44%)—7 of these patients were evaluated with PERCIST and 12 patients with RECIST 1.1.

### 3.2. Adverse Events

The incidence of treatment-related adverse effects of grade 3 or higher was 44%. Higher-grade adverse events that were recorded in our patient collective were hypertension (any grade, 71%; grade ≥ 3, 26%), weight loss (any grade, 70%; grade ≥ 3, 7%), diarrhea (any grade, 44%; grade ≥ 3, 9%), and palmar-plantar erythrodysesthesia syndrome (any grade, 14%, grade ≥ 3, 2%) (Table 2). In case of grade ≥3 adverse events, immediate dose reductions by at least 4 mg lenvatinib daily were accomplished. No treatment-related deaths occurred in our patient collective. Persistent adverse events of any grade led to treatment discontinuation in 16% (*n* = 7 patients). Drug holiday due to adverse events lasted no longer than 3 days in any case.

### 3.3. Lenvatinib Dosage

Dose reduction due to adverse effects resulted in a mean lenvatinib dose of 14 ± 5 mg per day (median: 14 mg; range: 4 to 24 mg). Four patients had no dose reduction at any time (9%), while 39 patients (91%) underwent (multiple) dose reductions in the course of disease. The maximum tolerated dose was 4 mg in one patient (2%), 10 mg in 16 patients (37%), 14 mg in 18 patients (42%), 18 mg in 2 patients (5%), 20 mg in 1 patient (2%), and 24 mg in 5 patients (12%), respectively. One patient (nr. 22) had a temporary dose reduction with a consecutive increase to 24 mg lenvatinib daily again. Seven patients (16%) discontinued lenvatinib treatment due to severe adverse events.

**Figure 1:**
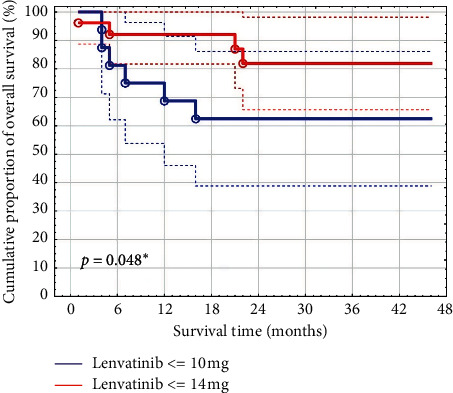
Overall survival with a daily dose of lenvatinib ≤10 mg and ≥14 mg (maintenance dose showed a statistically significant effect on survival).

The maintenance dosage of lenvatinib had a significant effect (*p*=0.048) on the overall survival of patients. Patients with a daily dose of ≤10 mg lenvatinib (*n* = 17) had an overall survival of 63% (95% CI: 39–86), while patients with a daily dose of ≥14 mg lenvatinib (*n* = 26) reached an overall survival of 82% (95% CI: 66–98) after 24 months, respectively (Figure 1).

### 3.4. TG Measurements

**Figure 2:**
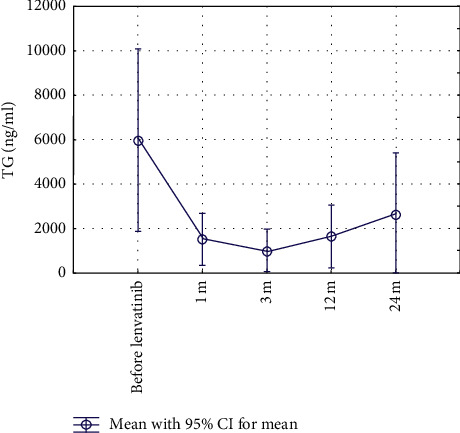
Change of thyroglobulin (TG) levels under therapy (1 m = 1 month, 3 m = 3 months, 12 m = 12 months, and 24 m = 24 months).

TG levels dropped by 75% after 1-month treatment with lenvatinib and reached a minimum after 3 months with a decrease by 98%. The change of mean TG levels before and after 1, 3, and 12 months of therapy was statistically significant (*p* < 0.05), as were changes between the first and third month of lenvatinib therapy (Table 3, Figure 2).

No statistical difference in TG levels (baseline vs. 12 months) could be found in patients with a daily dose of ≤10 mg lenvatinib and in patients with a daily dose of ≥14 mg, respectively.

## 4. Discussion

The clinical course of metastatic DTC shows clinically relevant variability with a range from patients with stable disease over many years to those with rapidly progressing disease and OS rates in the range of months upon diagnosis in least differentiated forms [10]. Real-world data are therefore of particular importance in RAI-refractory TC since they provide evidence on treatment efficacy and tolerability. The appropriate use of an anticancer drug in metastatic RAI-refractory TC in clinical routine should closely relate to the patient population tested in phase-III registration trials. This was true in our retrospective study in RAI-refractory TC patients exhibiting high rates of pulmonary (86% vs. 87%) and bone metastases (35% vs. 40%) when compared to the SELECT study [18, 23].

In the group of patients with progressive RAI-refractory DTC, lenvatinib significantly prolonged PFS to a median of 18.3 vs. 3.6 months in the placebo group [18]. In our analysis, the median PFS has not yet been reached. Seventy-one percent (95% CI: 56–87) of our patients showed PFS after 24 months, and OS after 24 months amounted to 74% (95% CI: 60–88). Notably, our findings relay on a longer observation period than the registration study with a median duration of treatment with lenvatinib of 27.6 months as compared to 13.8 months in the registration trial [18].

In our study, the patient population was treated nearly four times longer than in other real-life studies in Europe and Japan [24–28] (Table 4).

OS was notably high in our patient collective, in spite of consisting of an elder population with extensive metastatic disease, including hepatic and brain metastases. Thereby, patients with brain metastases exhibited a worse outcome with a median OS of 12 months, while this was not seen in patients with liver metastases with a median OS of 32 months.

At the time of data cutoff, 10 patients (23%) died due to progressive disease, and 7 patients (16%) discontinued with lenvatinib, and the remaining 26 patients (61%) were still on lenvatinib treatment.

Iodine deficiency was highly prevalent in the alpine region of Salzburg in Austria though its incidence was decreasing after the introduction of iodine supplementation [29]. Iodine deficiency has been associated with an increased proportion of FTC which might explain the predominance of FTC patients in our study (61%) as compared to SELECT with 38.7%. However, in agreement with the SELECT data, we found no impact of tumor histology on survival.

Every patient in our collective exhibited any form of adverse events of any grades, among which hypertension was most commonly seen (71%, 8 patients had grade 1, and 11 patients each had grades 2 and 3, respectively). When considering grade 3 or 4 toxicities, only hypertension was common, but less frequently seen than in the SELECT study (26% vs. 41.8%) [18].

Notably, a subanalysis of the latter demonstrated comparable efficacy in elderly subjects with a median age of 71 years in the presence of an increased toxicity profile [30]. The constellation of highly frequent adverse events in the presence of less high-grade toxicity might be associated with the finding of reduced maintenance dosages of lenvatinib employed throughout the observation period in our elder patient population. Decreased tolerability of any anticancer therapy might induce an earlier dose reduction in clinical routine than in clinical trials with their tight schedule of follow-up visits.

Weight loss (70%) was also more common in our study population which might be due to the longer median observation period than in SELECT with a reported incidence of 46.4%. Interestingly, weight loss was not always linked to decreased appetite, noted only in about 23% of our patients (vs. 50.2% in the SELECT study). The reasons for weight loss are multiple and might relate to impaired intake due to dry mouth or painful food intake, clinically relevant proteinuria, and/or a catabolic state due to tumor cachexia.

Interestingly, very recently, a considerable proportion of RAI-refractory patients treated with lenvatinib has been shown to develop symptomatic biliary disorders [31]. These conditions might also contribute to significant weight loss. However, in our patient cohort, no patient presented with an onset of symptomatic biliary disease.

Persistent adverse events of any grade led to the discontinuation of treatment in 7 patients (16% vs. 14.2%) in our collective. Any form of dose reduction was noted in 91% of our patients, which was more common than in the SELECT study with 67.8%. The mean maintenance dose of lenvatinib was 14 mg per day as compared to 17 mg in the SELECT study. Dose reduction was mostly required during the first year of treatment, which is consistent with findings from both original and updated analysis of the SELECT patients [23, 32], in which the percentage of patients with grade ≥ 3 lenvatinib-related adverse events increased by less than 5% after 3 years of follow-up [23]. No new treatment-related deaths were reported in the updated analysis. SELECT and our data suggest that lenvatinib might be effective in patients with progressive RAI-refractory TC at lower than the standard dosage recommended for the use in RAI-refractory DTC.

Of note, lenvatinib has also been approved for the first-line treatment of advanced hepatocellular carcinoma (HCC) [33]. Interestingly, the maximum tolerable dose (MTD) for lenvatinib in HCC patients is significantly lower compared to RAI-refractory TC patients with a daily dose of 12 mg/day for bodyweight ≥60 kg or 8 mg/day for bodyweight <60 kg [34]. The majority of HCC patients treated with lenvatinib needed dose reduction or drug withdrawal due to treatment-related adverse events [33]. Animal studies suggested that lenvatinib is predominantly metabolized in the liver and subsequently excreted via urine and feces [35]. Unmetabolized lenvatinib in urine and feces only accounts for 2.54% of the administered dose, hinting at a crucial role for liver metabolism in the elimination of lenvatinib [36]. In fact, HCC patients show a similar plasma concentration after multiple doses of 12 mg/daily lenvatinib due to impaired liver function, like patients with solid tumors treated with multiple doses of 25 mg/daily lenvatinib, resulting in similar areas under the curve (AUCs) [37]. In HCC patients, the AUC of lenvatinib is a reliable prognostic factor for dose reduction and occurrence of adverse events [34]. However, to the best of our knowledge, there have been no data on lenvatinib pharmacokinetic data in patients with TC, only data on solid tumors [38, 39].

An ongoing comparison phase-2 trial of lenvatinib (E7080) employing a starting dose of 18 mg versus 24 mg daily in RAI-refractory DTC patients addresses efficacy and safety profile in relation to dosage (211 study; NCT02702388).

Laboratory measurements of TG levels were used to assess disease progression biochemically in addition to standard imaging procedures. We detected a significant reduction in TG levels within a month by 75%, which is in agreement not only with SELECT but also other trials with a range from 82 to 86% [40, 41]. A reduction could also be found in patients with low maintenance doses of lenvatinib (≤10 mg daily). In an exploratory biomarker analysis of SELECT [42], the magnitude of change in TG levels appears to be associated with the objective response [42]. In our studies, a decrease in TG levels was also observed in patients with stable disease (SD) or progressive disease (PD), stressing the importance to validate other biomarkers [42] and/or imaging techniques for the assessment of treatment response.

Transient TG oscillations in the course of treatment were a frequent phenomenon that may not necessarily reflect morphologic tumor progression due to Werner et al. [41] and might be related to dose reductions or interruptions.

### 4.1. Limitations

A limitation of the study was the retrospective nature of our data collection. We included all histological subtypes of DTC in our clinical experience, as well as patients with ATC, who were given lenvatinib as an individual medical treatment due to tumor board decision.

Clinical, biochemical, and imaging analysis were performed at regular intervals though on behalf of the individual clinical judgement of the responsible physician. However, all centers applied similar protocols for the standard of care of DTC. In Austria, nuclear medicine departments are specialized for the management of thyroid cancer patients favoring similar diagnostic and therapeutic approaches which has also been published in a national consensus on the management of DTC by the use of TKI [6]. Notably, no patients were lost for follow-up indicating a high level of patient adherence.

It is another limitation of this study that either a PERCIST- or RECIST 1.1-based imaging analysis was used to evaluate tumor progression throughout different study centers. Moreover, PERCIST or RECIST 1.1. data were not available in a subset of study patients though the evaluation of the course of the disease in the respective center always comprised clinical, laboratory, and imaging-derived information.

Though FDG PET/CT has been shown to provide diagnostic and prognostic information in recurrent DTC due to its potential to identify RAI-refractory metastasis [43, 44], this technique is not uniquely recommended and employed in clinical routine.

Dose reductions were clinically required and performed based on the clinical judgement of the responsible specialist with differences regarding to their extent and duration and possible effects on the efficacy of the treatment.

## 5. Conclusion

Lenvatinib therapy showed sustained clinical efficacy in patients with metastatic RAI-refractory DTC in terms of year-long (progression-free) survival in clinical practice in Austria even when using lower reduced maintenance dosages. When employing lower dosages, toxicity is similar to the profile in controlled clinical trials, but mostly of lower grade, manageable, and clinically tolerable to the patient. The biomarker TG parallels the rapid clinical response during the first weeks of treatment. There is an obvious need for further controlled trials to assess the efficacy, tolerability, and toxicity profile of reduced daily dosage treatment regimen of lenvatinib in comparison to the standard dose of 24 mg recommended in RAI-refractory DTC.

## Figures and Tables

**Table 1:** Patient characteristics.

	*N* = 43
Median age (years, range)	70 (39–91)
Female (*n* (%))	18 (42)
Histologic subtype (*n* (%))	
Papillary	10 (23)
Follicular	26 (61)
Poorly differentiated	4 (9)
Anaplastic and follicular	2 (5)
Anaplastic	1 (2)
Metastatic lesions (*n* (%))	
Pulmonary metastases	37 (86)
Bone metastases	15 (35)
Lymph node metastases	32 (74)
Liver metastases	7 (16)
Brain metastases	5 (12)
Soft tissue metastases	11 (26)
Prior radioiodine therapy (*n* (%))	
None	2 (5)
One	10 (23)
Two	13 (30)
Three	7 (16)
Four	6 (14)
More than four	5 (12)
Median radioiodine activity (MBq, range)	11100 (1780–41277)
Prior TKI therapy	
Yes	8 (19)
No	35 (81)

**Table 2:** Overview of adverse events.

Adverse events	All grades (%)	Grade 1 (%)	Grade 2 (%)	Grade 3 (%)	Grade 4 (%)
Hypertension	71	19	26	26	0
Decreased weight	70	30	33	7	0
Fatigue	59	33	26	0	0
Proteinuria	48	29	19	0	0
Diarrhea	44	26	9	7	2
Decreased appetite	23	14	9	0	0
Asthenia	21	19	2	0	0
Palmar-plantar erythrodysesthesia syndrome	14	12	0	2	0
Electrolyte derailment	14	12	2	0	0
Vomiting	7	7	0	0	0
QT-prolongation	5	5	0	0	0
Mouth dryness	5	5	0	0	0
Liver dysfunction	2	0	2	0	0

**Table 3:** Thyroglobulin levels (mean ± SD) at different time points.

Thyroglobulin levels (ng/ml)	Mean	SD
Max TG (before therapy)	5,977.72	13,692.36
1 m	1,515.15	3,550.66
3 m	980.51	3,133.72
12 m	1,640.60	3,566.92
24 m	2,643.75	5,257.11

**Table 4:** Real-life studies from Europe and Japan.

Country	*N*	Age (median; years)	Gender (m/f)	Lenvatinib dose median (min–max)	Median duration of lenvatinib therapy (months)	PFS (median; months)	OS (median; months)	Observation period (median; months)
Italy [22]	94	60	50/46	19.2 mg (10–24 mg)	5.9	10.8	23.8	n.m.
France [23]	75	65	42/33	20 mg (n.m.-24 mg)	6	10	Not reached	7
Austria	43	73	25/18	14 mg (4–24 mg)	27.6	Not reached	Not reached	34.6
Japan [24]	42	66	12/30	Mean 10 mg (4–24 mg)	14.9	n.m.	n.m.	15.4
Netherlands [25]	39	72	20/19	18.6 mg (10–24 mg)	6.1	9.7	18.3	n.m.
Switzerland [26]	13	72	n. m.	n. m. (10–24 mg)	9.98	7.2 (estimated)	22.7 (estimated)	n.m.

n.m. = not mentioned.

## Data Availability

The data used to support the findings of this study are available from the corresponding author upon request. For specific data from the research locations, contact G. Rendl (Salzburg, g.rendl@salk.at), A. Becherer (Feldkirch, Alexander.Becherer@lkhf.at), C. Trummer (Graz, christian.trummer@medunigraz.at), H. J. Gallowitsch (Klagenfurt, Hans-Juergen.Gallowitsch@kabeg.at), and M. Raderer (Wien, markus.raderer@meduniwien.ac.at).
